# Development and validation of a timely and representative finite element human spine model for biomechanical simulations

**DOI:** 10.1038/s41598-020-77469-1

**Published:** 2020-12-09

**Authors:** Ibrahim El Bojairami, Khaled El-Monajjed, Mark Driscoll

**Affiliations:** Musculoskeletal Biomechanics Research Laboratory, Department of Mechanical Engineering, McGill University, 817 Sherbrooke Street West, Macdonald Eng. Bldg. Office #153, Montreal, QC H3A 0C3 Canada

**Keywords:** Biomedical engineering, Computational models

## Abstract

Numerous spine Finite Element (FE) models have been developed to assess spinal tolerances, spinal loadings and low back pain-related issues. However, justified simplifications, in terms of tissue decomposition and inclusion, for such a complex system may overlook crucial information. Thus, the purpose of this research was to develop and validate a comprehensive and representative spine FE model inclusive of an accurate representation of all major torso elements. A comprehensive model comprised of 273 tissues was developed via a novel FE meshing method to enhance computational feasibility. A comprehensive set of indirect validation tests were carried out to validate every aspect of the model. Under an increasing angular displacement of 24°–41°, the lumbar spine recorded an increasing moment from 5.5 to 9.3 Nm with an increase in IVD pressures from 0.41 to 0.66 MPa. Under forward flexion, vertical vertebral displacements simulated a 6% and 13% maximum discrepancy for intra-abdominal and intramuscular pressure results, all closely resembling previously documented in silico measured values. The developed state-of-the-art model includes most physiological tissues known to contribute to spinal loadings. Given the simulation’s accuracy, confirmed by its validation tests, the developed model may serve as a reliable spinal assessment tool.

## Introduction

Chronic Low Back Pain (LBP) prevails as a burdensome restrictive condition given its persistent apparent socioeconomic repercussions^1–4^. In industrialized societies, such as Canada and the United States, medical expenditures and consequential losses spiral up to $50 Billion USD annually^5,6^. Such incurred costs develop from the challenges present in the accurate assessments of LBP as a result of the broadness of its most common defined cause, sustaining both, mechanical and clinical instabilities^7–9^. In essence, the ability of the musculoskeletal system to perform its normal functions relies on its capability to maintain an upright structure, commonly known as ‘Spinal Stability’. In general, engineering stability pertains to a system’s ability to restore itself and maintain its initial state when perturbed. This in part aligns with the widely accepted definition of clinical spinal stability, perhaps best explained by Panjabi^7^ which attests that the spine maintains its three main functionalities which include protecting spinal cord and nerve roots, carrying loads and enabling motion. The deterioration in spinal stability results in numerous problems, most commonly, LBP which motivates the necessity to better understand the spinal system mechanism and pain pathomechanisms.

Due to the system’s redundancies involving different degrees of freedom, multiple input loads, complex joint-muscle connectivity and intricate soft tissues, assessing the musculoskeletal system has always been a challenging venture. In such a case, modelling presents itself as a plausible strategy to tackle elaborate systems such as the spine^10–12^. One conceivable, widely used, modelling tool is the Finite Elements Method (FEM), which was first proposed by Richard Courant in 1922^13^. Since then, numerous biomechanical FE models have been developed^14–17^ and used in various applications. One interesting example pertaining to spine biomechanics is the model developed by Driscoll et al., in which the authors discussed the adverse effects of spinal deformities, considered to be on the end of the instability spectrum, which conceived a platform to analyze novel spinal screw designs as a corrective treatment of such deformities^18,19^.

Numerous FEMs have been reported in literature to analyze spinal loading profiles, injuries, and its mechanical tolerances. Such models vary from a simple spine model which utilizes just a few elements^15,20,21^, to models detailing only the Vertebral Bodies (VB) and Intervertebral Discs (IVD)^14,22,23^, to more involved models that include simplified muscle geometries^24–26^. Other studies developed more physiologically realistic models by including the effects of Intra-abdominal Pressure (IAP) by using force vectors without the actual inflation in the diaphragm/abdomen^16^. However, this may tend to overestimate IAP values in certain areas due to the imbalance resulting from a fixed value of a varying fluidic IAP effect which changes with the muscular contraction. One of the most involved studies of including IAP was developed by Dietrich et al.^27^. In their study, they modelled the bones, cartilages, discs, ligaments, and muscles as non-linear anisotropic viscoelastic material, and IAP as an incompressible fluid embedded in a closed cavity, resulting with 2640 elements and 13,107 algebraic equation^28^. Similarly, Arjmand et al. developed an FE model based on mathematical representations which were constructed by Daggfeldt and Thorstensson^29^. Specifically, they represented the abdominal cavity as being encapsulated by three membrane layers to achieve a 7.35 mmHg baseline IAP elevating to a maximum of 29.627 mmHg IAP value during a partial Valsalva maneuver^30^.

In addition to the IAP, the pressure build-up in the spinal muscles, or in any other skeletal muscle to that matter, upon contraction (known as the muscle-balloon effect)^31^, is believed to play a role in spinal stability as put forth by the authors of the present study. As such, research efforts have been carried out to understand the mechanics of skeletal muscles, including their Intramuscular Pressure (IMP), and their overall contribution to human locomotion. Such a task is highly challenging due to the fact that numerous skeletal muscles, tendons, and joints interact together to produce a specific movement^32^. A well-developed and validated IMP-based FEM of a single muscle was put forth by El Bojairami and Driscoll^33^, in which the authors realized the existence of linear correlation between muscle forces and IMP. However, to the authors’ knowledge, incorporating the IMP in a full spine model has not yet been performed or studied.

One may argue that a reliable spine model should potentially incorporate all loading-assistive tissues attached to the spine^34–37^. That is, in addition to the spinal VBs, IVDs, IAP, and IMP effects, there remains an interest in including the ligamentous system that is believed to provide passive stiffness to the spine structure at limiting positions. In an interesting study, the ligamentous system in combination with the fascia, especially the Thoracolumbar Fascia (TLF), was examined by Gracovetsky through static computations^37^. It was suggested that their mechanical properties allow storing sufficient energy to permit the spine to overcome the extensive forces applied by the muscles. This was further developed by Moorhouse and Granata who observed that the tensile force applied by the spinal muscles, when resisting an applied flexion force, increases the stiffness of the spine^34^. In a complementing study, El-Monajjed et al. simulated the variational effects of the IMP and IAP in a 2D TLF model. They suggested that the IAP tended to realize a rather balancing role within the body during asymmetric postures^38^. Thus, the inclusion of all the aforementioned effects in a comprehensive spine FE models to examine the influence of such tissues would potentially permit performing detailed investigations.

Other reliable spine FE models tailored towards VBs and IVDs only, are abundant in literature, which like most models include some level of physiological simplification^17,39,40^. Such studies provide a great deal of insight into spinal loading, albeit the predicted values may be over-estimated in comparison with empirical data. In a well-executed study, sixteen senior groups that perform spinal investigations collaborated to compare 8 FE models of the lumbar spine (5 lumbar vertebral bodies and their 4 corresponding IVDs)^17^. Distinctions between the models were present such that Little et al. and Goel et al.^39,41^, included only the cylindrical part of the vertebral bodies with no spinous processes while Chen et al.^42^ included cartilages between vertebral bodies. In contrast, the other models^40,43,44^ represented the actual anatomical shape of the vertebral bodies and the IVDs. Furthermore, Schmidt et al.^40^ included the representation of muscles through simplified geometries, while the other 7 models modelled the muscles’ effect as vector forces. All other fascia, tendons, IMP, or IAP effects were excluded from these studies. For the first part, pure bending of 7.5 Nm was applied in all anatomical planes and all FE models simulated a maximum difference of 5° rotation which aligns with in-vitro data (median: 17°, range 11°–22°). Additionally, facet joint forces were predicted under the same loading condition. All FE models reported approximately 38 N in extension, 14 N in lateral bending, and 60 N in axial rotation; however, forces were considerably different between the models under flexion^17^. In conclusion, the comparative study suggested that combining different FE models improves the prediction to estimate biomechanical parameters, especially if such models individually involve different simplifications and assumptions.

It is evident that predictions conceived via FE analysis may not be worth interpreting unless the model was verified and validated. Essentially, a model is said to be validated if the results it predicts matches experimental observations^17^. Verification assesses the numerical accuracy of the underlying model to ensure that the computational model output properly represents the solutions to the corresponding mathematical equations^45^. Sensitivity, on the other hand, is the process of ensuring that the model is robust, i.e. predicts repeatable results, upon changing main input parameters such as the mesh size and quality^46^.

Thus, the purpose of this paper was to develop a detailed 3-dimensional comprehensive finite element model of the spine integrating the effects of IMP, IAP, and TLF. A series of tasks were executed via a diversified validation process to validate the model segment by segment against multiple published studies. In addition, a novel meshing technique was developed and utilized to compensate for the inevitable exceedingly high element count given the inclusion of the VBs, IVDs, all major spinal muscles with their IMP, IAP, connecting tendons, and TLF.

## Methods

### Creating the base model

The developed spine FEM was based on MRI-scans acquired from an anatomography; a database of 3D MRI-based human body parts, namely, “BodyParts3D/Anatomography”. The model of interest included the VBs from T_1_ to S_2_ joined together by IVDs. The major spinal muscles comprised of the longissimus, multifidus, psoas major, and lateral intertransversarius and were designated as force generators. Soft tissues, mainly the TLF and tendons, were also included to transmit muscle forces as well, due to their critical role of storing excess forces and stresses. Lastly, the IAP was further integrated modelled within a pressurized cavity enclosed by the abdominal muscles.

The acquired MRI-based model components were initially organized then processed into CAD supported files in SpaceClaim (V19.1, Concord, Massachusetts) to achieve a full-scale model consisting of a total of 302 parts. Numerous modules within the FE software, ANSYS (V19.1, Canonsburg, Pennsylvania), were employed to build the project. The detailed model of each part’s behavior and mechanical properties is described in the sections below.

### Volumetric bodies

The base model consisted of 17 VBs (12 thoracic and 5 lumbar vertebrae) linked by 16 IVDs. For the scope of this model, the cervical spine was omitted given its minimal relative effect on the employed model using the established series of tasks defined within this study. The base model components, i.e. the VBs and IVDs, were modelled as deformable volumetric bodies. In addition, tendons conveying muscle forces, and the TLF which are believed to provide structural support to the torso, were similarly modelled as deformable volumetric bodies. The core difference between the components was the assigned material law to each part which dictates its range of motion, resilience under an applied load, as well as the ability to store excess stress subjected to the spine. Although vertebral deformations are minimal in comparison to the other soft tissues, modelling them as deformable bodies with representative material behavior was intended to ensure the model’s accuracy and serve in subsequent studies thereof.

**Figure 1:**
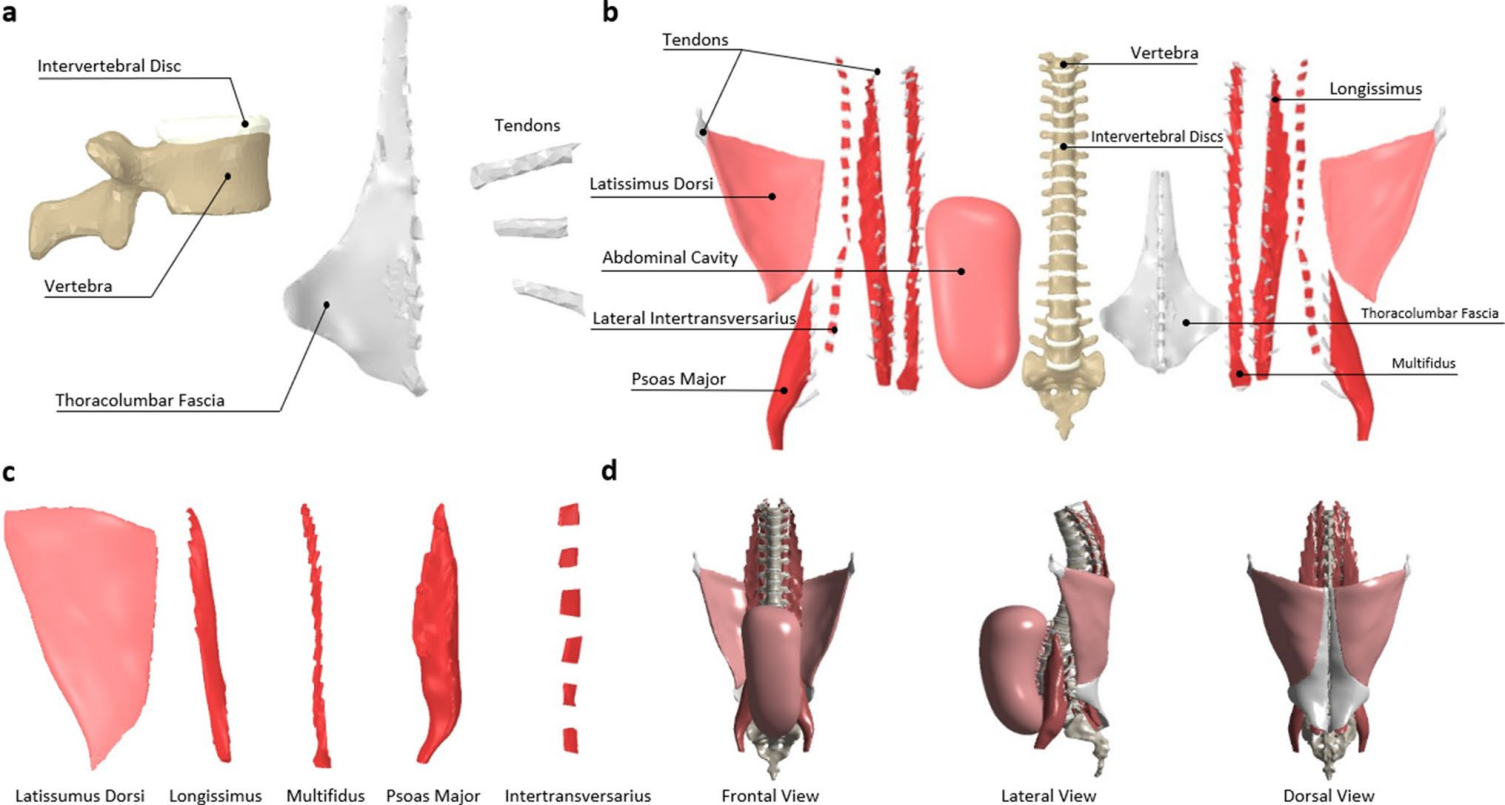
Finite element model of the spine produced via ANSYS Static Structural (v.19.1, Canonsburg, Pennsylvania, United States, https://www.ansys.com/). (**a**) Vertebral bodies, intervertebral discs, thoracolumbar fascia, and tendons modelled as volumetric deformable bodies. (**b**) Exploded view of all parts considered in the spine model. (**c**) Major torso muscles modelled as pressurized structures. (**d**) Frontal, lateral, and dorsal views of the full spine finite element model.

The geometrical representations of the various soft tissue were processed in SpaceClaim whereby sharp edges, low aspect ratio elements, and other unintended features that would otherwise increase the FE model complexity were eliminated. Each part was then transformed to a polygonal mesh with an edge size of 3 mm; a relatively computationally inexpensive element size that also enhances simulation accuracy. The detailed adopted mesh is further explained in “Adopted mesh” section below. An example of each of the aforementioned components is depicted in Fig. 1a.

Under external loading, detailed later as muscle forces, defined volumetric bodies may deform, translate, and rotate in all degrees of freedom. Such movements depend on the applied load, as well as their material behavior law, which also dictates possible changes in shape and volume under loading. Different material laws may be incorporated but for the scope of this paper, i.e. validating the spine model, material properties were adopted from the same investigations against which the model was indirectly validated, “Model validation” section below.

### Muscles and intra-muscular pressure (IMP) modelling

The presented model contains all major muscles that directly affect the spinal system. These include the longissimus, multifidus, psoas major, lateral intertransversarius, and latissimus dorsi (Fig. 1c). The longissimus, multifidus, and psoas major were included because, under spinal loading, they account for most of the load endured by the spine^47^. Similarly, the lateral intertransversarius were incorporated for their role of supporting the spinous processes of the torso under vertical compression/tension loading^48^. Finally, given the importance of including the TLF as discussed earlier, the latissimus dorsi was included as a result of its role in setting the TLF under tension^49^.

FEMs of human skeletal muscles are abundant in literature. However, as previously discussed, IMP is believed to play an important role during muscle contraction, and a novel accurate model that relates muscle forces to IMP, taking into account the actual muscle architecture, shape, and geometric features, has been previously explored^33^. As such, the procedure of building fluid–structure finite element field, by filling the muscle shell with hydrostatic pressure elements (HSFLD242 elements available in ANSYS) has been used for the present muscles. In short, the finite elasticity formulation started with the displacement field of the muscle shell. Since muscles are inherently incompressible, a displacement field would perform poorly in describing the whole muscle’s biomechanics due to a phenomenon commonly referred to as interlocking. This forces the researcher to build a two-field variational formulation to describe this fluid structure interaction of a muscle based on the principle of minimum potential energy. That is, to avoid the volume-locking phenomenon, in addition to the displacement field describing the muscle’s shell behavior, a hydrostatic pressure field is linked from the inside. For an incompressible Neo-Hookean material, the stored energy function, as formulated in^50^, is described by:

$$ \widetilde{W}_{C} \left( {{\varvec{X}},\overline{\user2{F}},\overline{p}} \right) = - \frac{\mu }{2}\left( {\overline{\user2{F}}:{\varvec{F}} - 3} \right) $$

Thereafter, by definition of the Cauchy stress tensor, the hydrostatic pressure is given by:

$$ p = \overline{p} - \frac{1}{{3\det {\varvec{F}}}}\frac{{\partial \widetilde{W}_{C} }}{{\partial {\varvec{F}}}} :{\varvec{F}} $$

This was numerically solved via ANSYS by custom coding the pressure field. Enclosed within each muscle’s shell, the HSFLD242 elements share a hydrostatic pressure node (HDSP) allowing to extract IMP results, as well as introducing this pressure to stimulate muscle contraction. The novelty of such a procedure lies within the ability of relating muscle forces to their IMP, simulating one effect as a result of the other, and accounting for physiological muscle lateral growth under contraction.

### Intra-abdominal pressure (IAP) modelling

The importance of including the IAP effect stems from investigations towards understanding its exact role. Bartelink^51^ suggested that the activation of abdominal muscles translates into an IAP, which stabilizes the lumbar spine by providing an opposite unloading effect. The present model focuses on this effect, rather than its activation. As such, IAP was modelled as a pressure build-up enclosed by an abdominal cavity, defined by the abdominal muscles, soft tissues reaching the frontal part of the VBs, and the diaphragm from the top. MRI scans of existing organs in that region were processed and their surfaces were traced to create a model of the abdominal cavity. Thereafter, similar to the adopted muscle model strategy, IAP was introduced as a hydrostatic pressure effect building up in the abdominal cavity by merging the cavity’s shell elements with HSFLD242 elements enclosed inside. This cavity, along with the full spine model, is shown in Fig. 1b,d.

Due to the limited published data on the abdominal wall mechanical behavior, material properties were extracted from the results of the experimental study conducted by Song et al.^52^, performing ultrasound measurements of the abdominal wall. This resulted with a Young’s moduli: $E_{transverse} = 42.5 \,{\text{KPa}}; E_{sagittal} = 22.5 \,{\text{KPa}}$ and an average abdominal wall thickness, defined by the abdominal muscles, of: $t_{wall} = 9.7 \,{\text{mm}}$.

### Adopted mesh

A common problem in the FE method entails a trade-off between accuracy and computational feasibility or speed. Less variability and a higher reliability results generally require: (1) a representative graphical model, (2) accurate description of material behaviors, and (3) a full-scale smooth mesh that captures all model features. For a representative benchmark spine model, the first two objectives were closely met as described above. The challenge remained to create a smart, novel, computational mesh that would not elongate simulation time. For reference, a conventional 3 mm size mesh resulted with over 0.6 million elements, with approximately 311 linear and nonlinear contact objects between the modelled parts. The simulation of a trial spine under 100 N flexion would take months to solve. This would not be feasible with the aim of creating a reference spine model to be implemented in the medical field.

**Figure 2:**
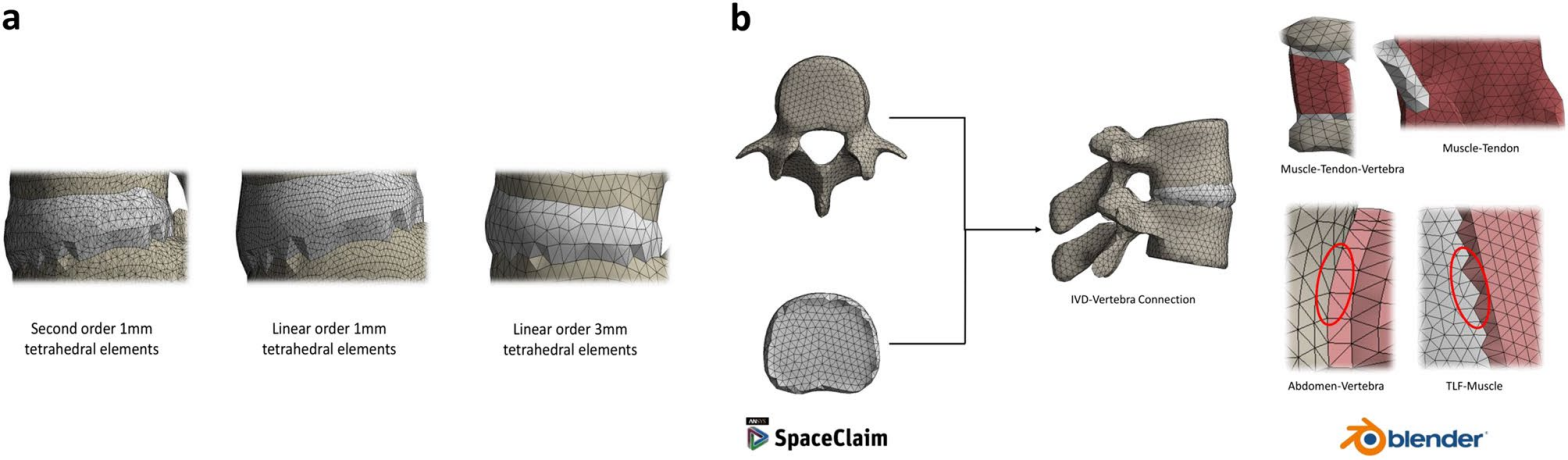
Finite element model produced mesh. (**a**) Three different meshes explored in the sensitivity analysis. (**b**) Adopted meshing technique showing conformity across contacting objects. Meshes were produced via the help of ANSYS SpaceClaim and ICEM CFD software packages (v.19.1, Canonsburg, Pennsylvania, United States, https://www.ansys.com/) as well as Blender (v.2.83.5, Netherlands, https://www.blender.org/).

Hence, this study adopted a novel meshing technique, whereby all FEM fixed contacts computations (applying nonlinear constraints and penalty functions) would be eliminated, with all contacting bodies, having adjacent surfaces, sharing the same nodes. This would not only relax the simulations and produce more accurate results, but would also result with better approximations as conventional penalty functions would not be applied anymore^53^. The idea was to create a unitary mesh whereby under any loading condition, over contacts that are physiologically fixed, the load would distribute and travel among nodes with no barrier of iterating the mathematical equations of the contacts. This was done by leveraging ICEM CFD component of ANSYS to create shell meshes for the muscles and abdominal cavity, which were then filled with HSFLD242 fluid elements prior to simulation. Further, the Multizone Method in ANSYS meshing tool tetrahedral volumetric elements for the other organs. An intermediate stage of exporting adjacent parts into Blender, a free and open-source 3D computer graphics software, was then used to merge adjacent nodes on contacting bodies together. After iterating all present bodies in the model, all mesh files were then collected by a single ANSYS mesh component to run a final compatibility check before proceeding with simulating the model. Figure 2b illustrate this meshing procedure.

This time-consuming step was justified as the final version of the model took roughly 3 min to solve, though comprised of 398,217 elements and 547,380 nodes.

### Model validation

Indirect validation of comparing the model’s results against readily available literature data was carried out. Due to the model’s novelty of including major tissues, IAP, and IMP effects, a complex and diversified sensitivity case-study was performed. Each component within the developed model was validated against appropriate published data in efforts to conclude on the overall validity of the present model. As such, the following validation tests were conducted:

#### Muscles and enclosed pressure

**Figure 3:**
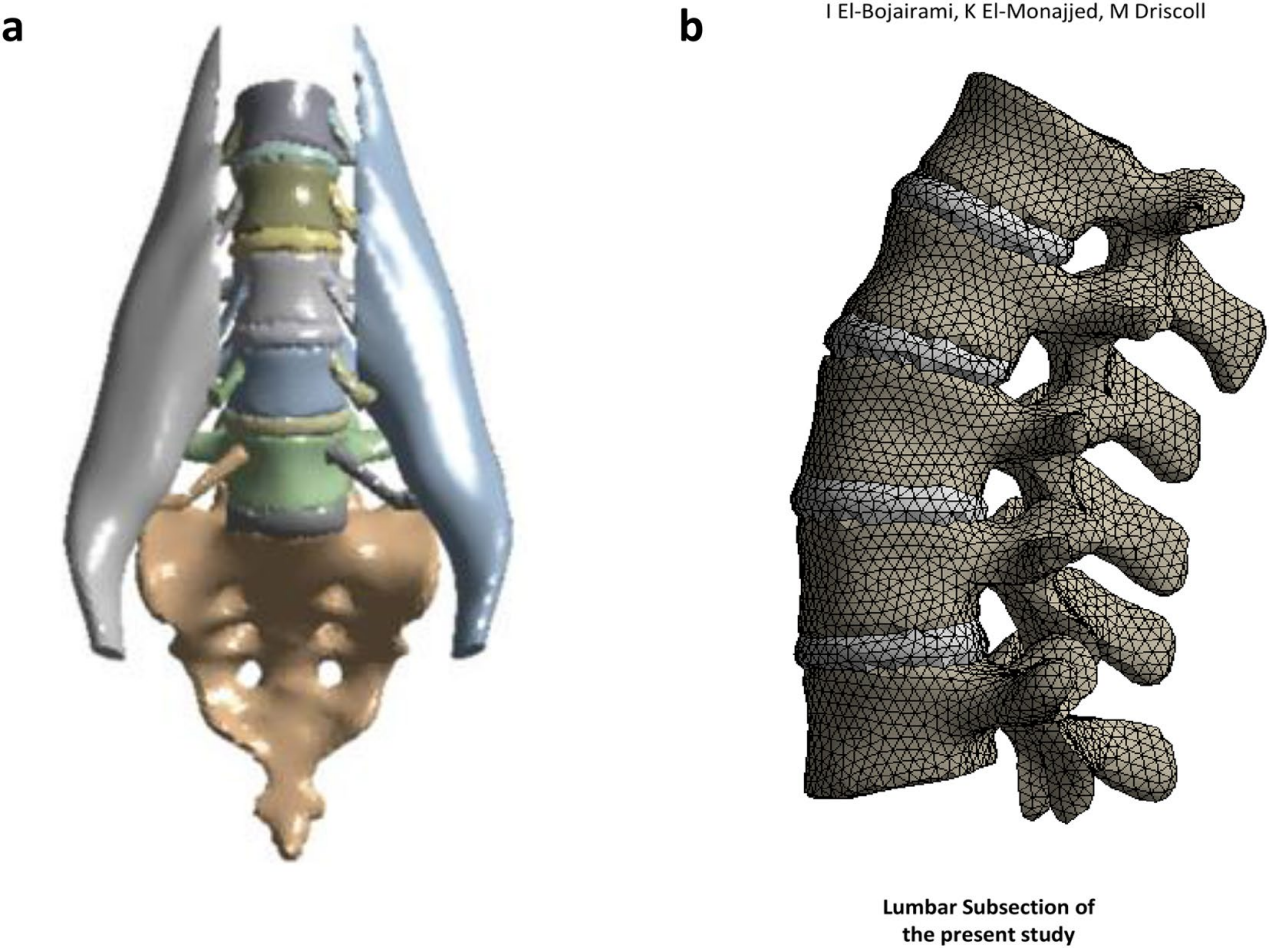
Lumbar spine finite element model. (**a**) Lumbar spine and psoas major muscle model isolated to perform the muscles and enclosed pressure validation. (**b**) Lumbar spine finite element model isolated to match the models against which validation was performed^17^ (Models were developed using ANSYS, v.19.1, Canonsburg, Pennsylvania, United States, https://www.ansys.com/).

The modelling of skeletal muscles as fluid-filled structures resulted in a linear direct correlation between muscle forces and IMP. A comprehensive mesh, material behavior, and tendon stiffnesses sensitivity analyses suggested a robust model^33^. Following the linearities of the model, expanding the developed model to other skeletal muscles would maintain this IMP-force correlation, resulting with a valid modelling procedure. To investigate this, the spine-IVDs lumbar structure, with the psoas major muscle and its tendons, were isolated, as shown in Fig. 3a, and EMG measured psoas major forces^47^ were applied across each insertion point. Dirichlet boundary conditions were applied on L1 and the sacrum, thereafter, IMP was plotted against different force levels to explore the validity of the linear IMP-force correlation. Table 1 summarizes the adopted material properties used in this scenario^54–58^.

**Table 1:** Material properties used for the lumbar spine test.

Component	Material properties	Thickness
Vertebral bodies	E = 12 GPa; $v$ = 0.3^50^	–
Intervertebral discs	E = 42.7 MPa; $v$ = 0.499 (Incompressible)^49^	–
Psoas major muscle	E = 0.52 MPa; $v$ = 0.499 (Incompressible)^51^	2.73 mm^48^
Tendons	E = 1 GPa; $v$ = 0.499 (Incompressible)^52^	–

#### Lumbar spine

A detailed comparative study between eight, novel, different lumbar spine FEMs^17^, earlier detailed in the “Introduction” section, was used to validate the lumbar part of the present model. The lumbar part of the present model was isolated to mimic the structure of those published^17^ (Fig. 3b). Due to the wide range of material properties used, summarized in Table 1 of Dreischar et al., different material properties, being those used in the first scenario, were adopted for the present model. Thereafter, inverse validation was conducted, whereby the range recorded L_1_–L_5_ rotations in flexion–extension (i.e. 24°–41°), was applied while a reaction bending moment around the same anatomical plane was extracted from the FE model. This was done in order to investigate the resulting level of rotation with their applied 7.5 Nm bending moment. Using the same boundary conditions, Dirichlet conditions were also applied at the sacrum level, preventing any displacement in all degrees of freedom.

#### Intradiscal (IVD) pressure

Using the same lumbar model, under the same loading conditions and material properties, intradiscal (IVD) pressure values were recorded to be compared to normal published range. This was done by extracting the average normal stress recorded at the surface of the IVDs. Although, previously shown that this approach approximates the pressure build-up in a structure according to the principle of pressurized vessels under static condition^33^, a better IVD model that allows extracting the actual pressure within would provide more reliable results. As such, the fifth lumbar IVD was divided into its annulus fibrosis and nucleus pulposus components. The anulus fibrosis was modeled as a volumetric deformable object with a Young’s Modulus of E = 8 MPa and a Poisson’s Ratio of $\nu$ = 0.45^59^, while the nucleus pulposus was modelled as shell structure enclosed with hydrostatic HSFLD242 pressure elements with the shell assigned a 1 mm thickness^60^, young’s modulus: E = 1 MPa, and a quasi-incompressible Poisson ratio of $\nu$ = 0.49^17^. Thereafter, the pressure inside the fifth lumbar vertebra was extracted and compared to the normal stress approach.

#### Full spine validation

Lastly, the VBs and IVDs of the whole model were isolated to mimic the behavior and validate against a numerical model constructed in LifeMOD^61^. Under the same loading conditions, i.e., recorded flexion force range of 0 to 350 N, translation of VBs T_10_ to L_5_ was recorded and compared to Huynh’s findings. However, due to the wide difference between both models, with one being the inclusion of the actual muscles in the present model, another approach was conducted in efforts of validating the presented full spine model. This was done to ensure that the inclusion of all IMP, IAP, TLF, and the other soft tissues would result with a valid model.

The other approach consisted of applying all muscular contribution detailed by the EMG recorded data of Cholewicki et al. and Hansen et al.^5,47^ by the included muscle, thus simulating spine flexion caused by muscles contraction. Following this, the model’s extremities, including T_1_, were fixed and the load resulting on T_1_ was measured. Thereafter, according to Cholewicki and Hansen muscular proportions, muscle forces were varied until they resulted with the minimum applied forward flexion force of 50 N in^61^. Thereafter, muscle forces were increased in 5 increments and a similar curve of the resultant values of T_1_ load vs. T_10_–L_5_ vertebral translations was plotted and compared to the previous one. By doing so, the present model would ensure the incorporation of the additional effects resulting mainly from the IMP, IAP, and TLF contribution, rather than such effect being completely passive. Again, Dirichlet’s boundary conditions were also applied at the sacrum and the tendons attached to the latissimus dorsi. The material properties employed are summarized in Table 2^38,52,54–58,62^.

**Table 2:** Material properties used for the full spine validation test.

Component	Material properties	Thickness
Vertebral bodies	E = 12 GPa; $v$ = 0.3^50^	–
Intervertebral discs	E = 42.7 MPa; $v$ = 0.499 (Incompressible)^49^	–
Psoas major muscle	E = 0.52 MPa; $v$ = 0.499 (Incompressible)^51^	2.73 mm^48^
Multifidus muscle	E = 36.87 kPa MPa; $v$ = 0.499 (Incompressible)^58^	~ 4.5 mm^48^
Longissimus muscle	E = 36.87 kPa MPa; $v$ = 0.499 (Incompressible)^58^	4.03 mm^48^
Latissimus dorsi muscle	E = 36.87 kPa MPa; $v$ = 0.499 (Incompressible)^58^	~ 4.5 mm^48^
Intertransversarius muscles	E = 36.87 kPa MPa; $v$ = 0.499 (Incompressible)^58^	~ 1 mm^48^
Tendons	E = 1 GPa; $v$ = 0.499 (Incompressible)^52^	–
Thoracolumbar fascia	E = 450 MPa; $v$ = 0.499 (Incompressible)^57^	–
Abdominal wall	E = 25 kPa; $v$ = 0.45^56^	9.7 mm^56^

### Sensitivity case-studies

#### Model form validation

As suggested by ASME V&V 40-2018 guidelines^63^, model form validation is essential to ensure that the FE model closely captures the origin from which the model stems, being MRI scans in this case. Thus, it was essential to compare the computational mesh size to the original MRI scans size. For this, it was decided that the metric of comparison to be the volume in both cases. For simplicity, this analysis was done for the lumbar part only, i.e., L_1_–L_5_ with their IVDs.

#### Mesh sensitivity

With the adopted mesh strategy being one of the novelties of this study, it was essential to verify the accuracy of the adopted meshing procedure. As such, two other meshes were explored: (1) a linear tetrahedral mesh but with smaller element size, being 1 mm, and refined around sharp angles and edges using the pinch and inflation features in ANSYS and (2) a second order tetrahedral mesh, with one element composed of 10 nodes instead of 4 in the case of linear tetrahedrons, with 1 mm element size and properly refined as well (Fig. 2a).

For the purpose of comparison between those meshes, the scenario of “Lumbar spine” section above was repeated, recording the moment resulting from the levels of rotations for each of those meshes.

## Results

### Model validation

#### Muscles and enclosed pressure

**Figure 4:**
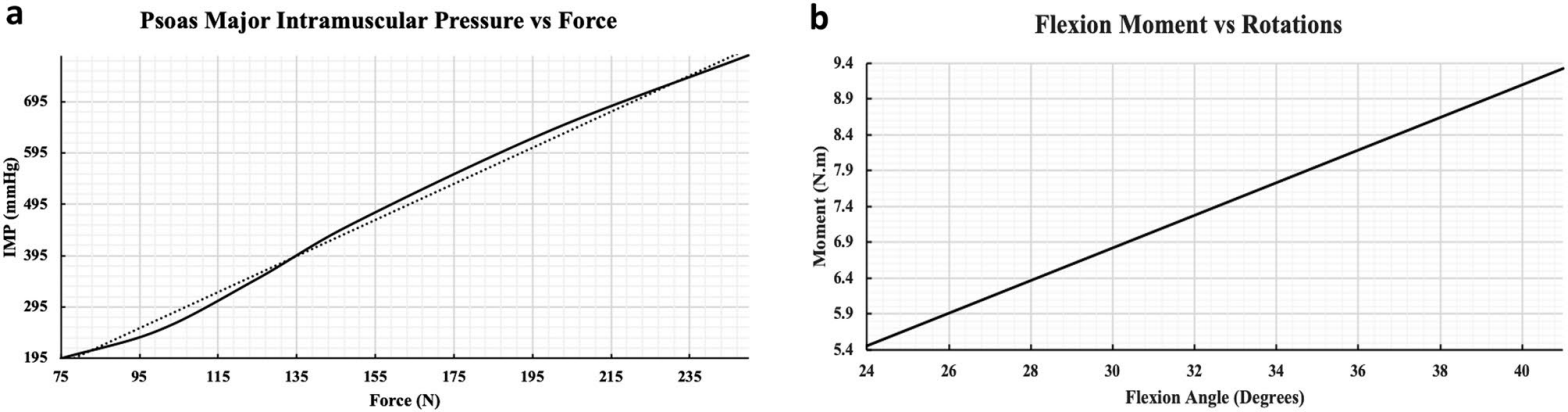
Intramuscular pressure and lumbar spine validation results. (**a**) Results showing the relation between muscle forces (N) and IMP (mmHg) extracted from the psoas major muscle. (**b**) Recorded bending moment (N.m) as a result of the lumbar spine elevation intensity (Degrees).

Forces of the Psoas Major (PM) previously measured via Electromyography (EMG)^47^, under the documented physiological condition, recorded an average of 249 N for the right PM and 275 N for the left PM under flexion, while 74 N for the right PM and 75 N for the left PM under extension. Simulating all possible scenarios between flexion and extension, the range of 75–275 N was applied in both PM muscles. The resultant force-IMP curve for the right PM muscle is shown in Fig. 4a. Results showed an approximately linear correlation (R^2^ = 0.995), between PM forces and IMP whereby the latter increased from 195 to 785 mmHg as the force increased from 75 to 250 N.

#### Lumbar spine

In the first task, the range of rotations of 24°–41° across the lumbar spine were applied as detailed in the “Methods” section. Thus, the resultant bending moments, defined by Eq. (1) as a special ANSYS command output, were recorded (Fig. 4b).

1 $$ M_{Bending} = F_{y} \cdot d $$

Such that: $M_{Bending}$: Bending moment about the transverse plane, $F_{y}$: In-plane reaction force (sagittal plane normal vector reaction force resultant), *d*: Moment arm defined as the distance between the reaction force and the parallel plane containing the model center of gravity.

When simulating the intensity elevation (i.e. 24°–41°) of lumbar spine in flexion, the bending moment recorded at the level of L_5_ linearly increased from 5.5 to 9.3 Nm. Similarly, the in-vitro experimentation^17^ showed that a $35 \pm 2$° degree flexion would be the result of a 7.5 Nm bending moment. Figure 4b shows an output simulation result of 33° flexion for the developed FE model. Furthermore, the present FE model predicted a follower compression load of 977 N compared to the 1000 N applied load they reported or a 98% correspondence.

#### Intradiscal (IVD) pressure

**Figure 5:**
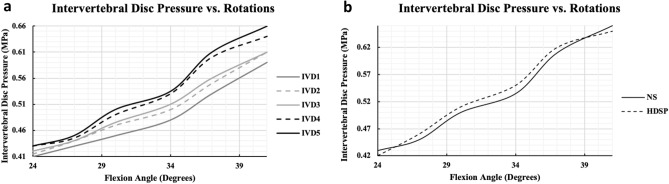
Intervertebral discs validation results. (**a**) Recorded lumbar intervertebral discs pressure as a result of spine flexion intensity, extracted via the average normal stress approach. (**b**) Verification of the normal stress approach via another two-state fluid–structure field using a hydrostatic pressure node.

Under the same loading conditions of the second task, i.e. increasing the flexion intensity from 24° to 41°, IVD pressure values in the lumbar spine increased from a range of 0.41–0.43 to 0.59–0.66 MPa (Fig. 5a) for all IVDs (i.e. 1–5). The data further showed an increasing pattern for both flexion/extension at any spinal level such that the intradiscal pressure value increases inferiorly to IVD_1_, with the maximum registered at IVD_5_. The data closely resembled the normal physiological ranges of intradiscal pressure of 0.4 to 0.8 MPa, during flexion/extension, as experimentally conducted by Wang et al.^64^ on 3 different subjects. Furthermore, the present data differed by 14% in comparison to the investigations conducted by Rolander et al.^65^ and Ranu et al.^66^. By tailoring the degree of flexion to attain a compressive load similar to thar applied by previous scholars, Rolander et al.’s case study showed an increase in intradiscal pressure from approximately 0.37 to 0.63 MPa, while an increase from a range of 0.3–0.4 to 0.6–0.8 MPa for the investigation of Ranu et al.

On the other hand, the approach of extracting IVD pressure as an average normal stress (NS) recorded at the surface of each IVD was compared to another more representative approach, as detailed in the methodology section, of modelling the IVD as a two-phase fluid–structure component and extracting the IVD pressure from the fluid model of the nucleus pulposus, from a hydrostatic pressure node (HDSP) assigned within. Figure 5b shows that both results overlap to a great extinct, with a maximum discrepancy of approximately 4% at 34° flexion.

#### Full spine validation

**Figure 6:**
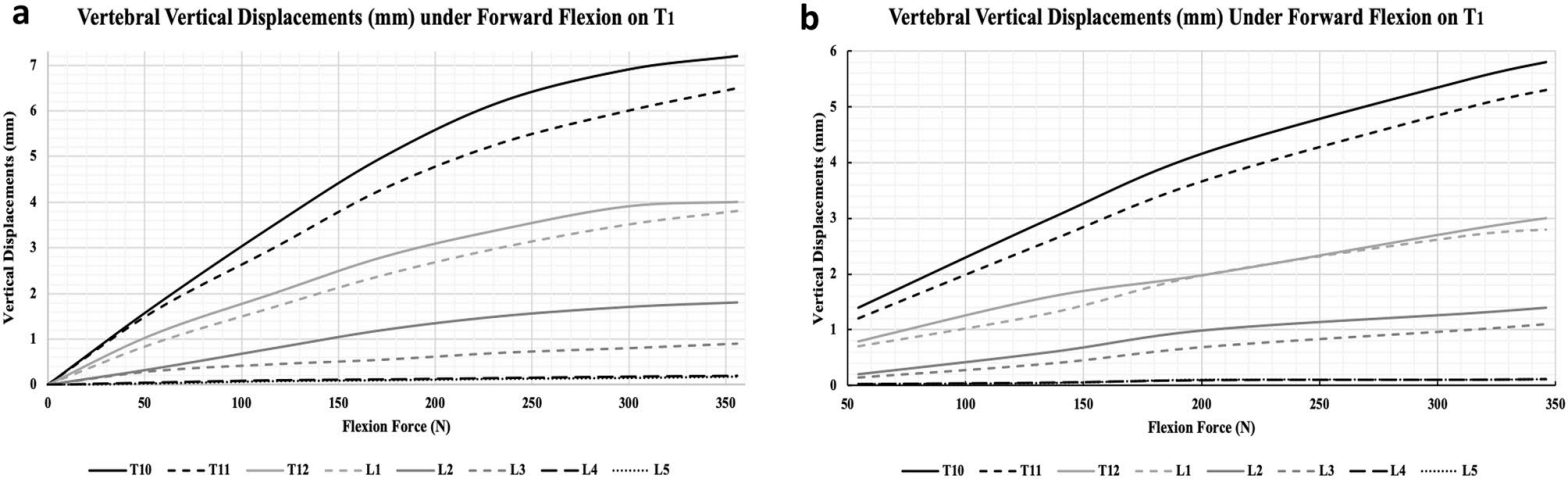
Full spine validation. (**a**) Recorded vertical vertebral displacements in response to an external flexion force applied on the first thoracic vertebrae. (**b**) Verification of the external applied flexion force approach via a muscles active contraction approach, whereby involved muscles actively contract to mimic the external flexion force.

The final step was to validate the full model with all its components and included effects. Initially, the spine included the VBs and IVDs only and a forward flexion of 0 to 350 N was applied on T_1_. The caudocranial translation of each VB was then extracted and plotted as shown in Fig. 6a. Vertebral displacements exhibited an increasing pattern, with a flexion force, at a higher rate for the superior VBs, recording around 7.1 mm and 6.5 mm displacement values for T_10_ and T_11_ respectively, at a 350 N flexion force. For T_12_ and L_1_, the displacements reported a 4 mm and 3.8 mm, while such displacements were minimal for the rest VBs, especially for L_4_ and L_5_ with a nearly null value under all flexion forces. Such results were highly correlated to those of Huynh’s et al.^61^, with a very small maximum discrepancy of approximately 6% recorded for T_11_ at a 300 N force.

In the second approach, previously reported muscle forces^47^ were first applied, as summarized in case 0 of Table 3, which resulted with a 382 N load on T_1_. Thereafter, muscle forces decreased in similar proportions until a T_1_ load of approximately 50 N was achieved, summarized by case 1. In subsequent cases, muscle forces increased in equal proportions, recording the resultant load on T_1_, until a maximum load of 346 N was obtained. It was also observed that muscle forces achieved a nonlinear increase in the T_1_ loading such that the more the muscle generated a force, the less the load increment was applied on T_1_.

**Table 3:** Muscle force inputs.

Muscle	Muscles forces (N)					
	Case 0	Case 1	Case 2	Case 3	Case 4	Case 5
Longissimus	210	30	85	120	185	198
Multifidus	71	15	30	45	65	71
Psoas major	275	40	100	140	235	260
Intertransversarius	25	5	10	15	25	25
Load on T1 (N)	382	54.4	136	203	314	346

Lastly, a similar curve of vertebral displacements was generated as shown in Fig. 6b. Interestingly, results followed the same trend as the previous findings, however, with a significant drop in the vertebral displacements at similar flexion forces. That is, vertebral displacements increased, with increasing flexion force, recording a maximum of 5.8 mm and 5.3 mm for T_10_ and T_11_ respectively, at 346 N. For T_12_ and L_1_, this was 3 mm and 2.7 mm, while such displacements were also minimal for the rest VBs, especially for L_4_ and L_5_ recording almost null values under all flexion forces. Furthermore, IAP recorded by the HDSP pressure node inside the abdominal cavity recorded an increase in IAP from 5 to 36 mmHg, and the force recorded at each TLF-VB connection showed a similar nonlinear increase from a minimum of 12 N to a maximum of 139 N resistive force.

### Sensitivity case-studies

#### Model form validation

**Figure 7:**
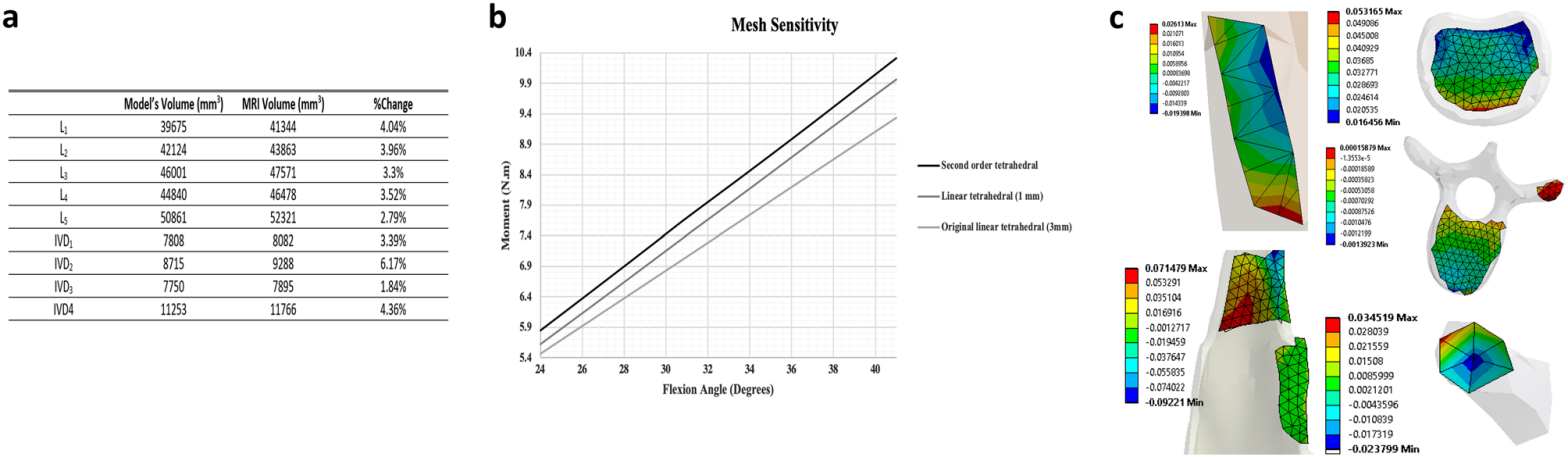
Model’s sensitivity case-studies. (**a**) Model’s form and development sensitivity analysis. (**b**) Model’s sensitivity to different meshes. (**c**) Maximum strain analysis (Conducted and extracted from ANSYS, v.19.1, Canonsburg, Pennsylvania, United States, https://www.ansys.com/).

Figure 7a summarizes the analysis conducted on the volumetric difference of the components within the lumbar part of the model. As depicted, the modelled mesh achieved an accurate representation of the original MRI scans with a maximum discrepancy of 6.17% registered for IVD_2_.

#### Mesh sensitivity

Simulating the lumbar spine section, as detailed in the methodology section, was conducted by increasing the degree of rotation from 24° to 41° such that the bending moment at the level of L_5_ increased from 5.6 to 9.9 Nm for the linear 1 mm tetrahedral mesh. Similarly, the value increased from 5.8 to 10.3 Nm for the second order 1 mm tetrahedral mesh (Fig. 7b). Compared to the original mesh, a maximum difference of 8.7% was registered between the original and the utmost refined second order mesh.

## Discussion

Advancements in the computational biomechanical field have paved the way for more accurate finite element model representations of the human torso and its biomechanical behavior. These FE models are usually constructed based on mathematical representations (i.e. considered case-specific), which disregard accurate geometric representations and may result in an over simplified model. Although for specific cases such approximations and assumptions are valid, such simplification leads such models astray from accurate physiological representation. For example, as of late, the effects such as the abdominal pressure, muscle pressure, and thoracolumbar fascia^38,67^ have been shown to play a role in spine biomechanics and hence their inclusion in biomechanical models may be warranted if one focuses their research thereon.

In accordance with this, a three-dimensional representative novel full-scale biomechanical spine FE model has been successfully constructed and validated. The model consisted of the thoracic and lumbar vertebral bodies, intervertebral discs, abdominal wall and its intra-abdominal pressure, thoracolumbar fascia, longissimus, multifidus, psoas major, latissimus dorsi, and intertransversarius muscles, as well as their accompanying tendons.

### Model development

Geometric modelling has been given attention to preserve MRI-scan features. That is, multiple modelling iterations were carried out to ensure each part was modelled accurately without significant loss in quality. Models of vertebral bodies and intervertebral discs were for example generated with a quality of at least 94%. This was achieved by directly faceting those parts and preserving the enclosed volume for deformable body modelling. The abdominal wall, on the other hand, required a relatively rigorous amount of effort as it required tracing the abdominal muscles, reaching the frontal side of the vertebral bodies and the diaphragm from above. This required collecting multiple opinions on the shape of the abdominal cavity and its biomechanical behavior. In addition, creating a representative model of muscles and their enclosed pressure was tedious which are substituted with force vectors in most developed models. As such, the first step towards building the present model was to create a tibialis anterior muscle model suited to predict muscle forces from its IMP and vice versa^33^. Nevertheless, as tendons are functional passive parts of the muscle structure, separating both parts with the intention of better modelling two-structure anatomy required the generation and faceting of some of the lost tendons. This resulted with some loss in accuracy, especially for the multifidus muscle due to its compact nature, as the volumetric difference increased to approximately 81% compared to the original MRI-based components. Yet, this was accounted for in the tendon model and ultimately parts of the muscle itself. Finally, the thoracolumbar fascia has been recently receiving growing attention and thus its inclusion was hypothesized to play a role in the force distribution within the model. However, the original model lacked the TLF’s connection to the vertebra, which required the integration of the faceted junctions at the vertebral dorsal portion. A similar 2-D planar model of the TLF has been validated and investigated by the authors^38^. Thus, the authors strived towards modeling all the components accurately.

Generating a representative computational mesh is designated as one of the most critical stages in FE modelling. Generally, researchers thrive to create a mesh that reliably reproduces results yet maintains a low complexity to carry out future simulations in a reasonable amount of time. Nevertheless, initial simulations were conducted using conventional methods which resulted with a high complexity overhead, requiring months to solve. Hence, the present model was meshed using a non-conventional novel technique. As described in the methodology, surfaces of contacting bodies were manually generated. As such, nonlinear contact computations were greatly reduced, whereby the load transmission mechanism between contacting bodies followed an explicit solution rather than iterative nonlinear as with the case of nonlinear ANSYS contact algorithms. That is, the entire spinal assembly became one structure, from the meshing perspective, for which the deformation of one object directly affects neighboring components. In human body mechanics, this is, in fact, recommended to ensure a homogeneous fluidic movement of all body parts. Therefore, in comparison, this novel meshing technique resulted with an enormous reduction for to achieve simulation results in just less than 3 min per simulation, depending on material properties and boundary conditions.

### Model validation

In satisfying computational reliability, validation plays a key element for any constructed FE model. Given the high number of components introduced into the model, validation of the entirety of the model in a single step was not possible to achieve specifically that no such model exists to the authors’ knowledge. As such, as detailed in the methodology section, a comprehensive case study was conducted in efforts of validating the model scoped to subsections.

#### Muscles and enclosed pressure

Modelling the skeletal muscles as pressurized structures provides a more accurate representation of muscle contraction, playing a significant role in both, intermuscular and intramuscular pressure. The validity of this modelling procedure stems from the proven valid representation of a two-state muscle, fluid–structure state, that was previously conducted^33^. However, it was still essential to investigate if muscle scaling would change the IMP-F relation. Under realistic muscle contractile force, collected from EMG data as illustrated previously, the Psoas Major (PM) muscles produced significant spine flexion under 275 N force, and extension under an opposing 75 N muscle force as suggested by Cholewicki et al.^47^. In fact, the linear correlation between muscle forces and IMP was consistently preserved (Fig. 4a). Such results were highly encouraging to model all other skeletal muscles, presented in the model, using the same procedure due to the proven potential of this accurate and representative muscle model in the FE field.

#### Lumbar spine

Lumbar spine models have received great attention rendering advanced investigations among biomechanical researchers. The effort done by Dreischarf et al.^17^, comparing eight different well-developed lumbar spine FE models, was of a particular interest as validating against all of those at once would leverage the accuracy and validity of the presented lumbar spine. Thus, in forward flexion simulations, results showed a linear increase in bending moment from 5.5 to 9.3 Nm with an increasing angle of flexion. Linearity, in this case, stems from the linear relation between force and bending moment, presented in Eq. (1). Additionally, an accurate forecast of 7.5 Nm occurred at flexion angle of 33° (Fig. 4b). In their investigation, in-vitro results showed that a 7.5 Nm moment would be the result of $35 \pm 2$° flexion. Nevertheless, with the current approach of applying the range of flexion and measuring the resultant bending moment, their applied follower load would be a result rather than an input in the current investigation. As such, retrieving the compression load at the level of L_5_ resulted with a 977 N load mimicking their 1000 N applied follower load. The main reason for simulating lumbar flexion this way is the fact that one crucial particularity of the full model is the inclusion of the major spinal muscles. That is, for the accurate representation of muscles producing spine movement, it was of interest to minimize other approximations that would substitute any muscular effort, mainly follower loads and muscles contribution modelled as force vectors.

Overall, the achieved results proved the high accuracy of the present FE model of the lumbar spine, combined with the reported material properties, when compared to the previously well-developed lumbar spine models in the literature.

#### Intradiscal (IVD) pressure

As described in the introduction, low-back pain is often associated to a malfunction with the lumbar spine associated with an excessive pressurization of the lumbar discs. Standing as one of the highest causes leading to disability^68^, intradiscal pressure data in conditions of low back pain cases is widely available. This permitted additional validation of the lumbar spine, mainly investigating the accuracy of the spine model to predict IVD pressure.

In the first scenario, under the normal range of flexion/extension explained in the *lumbar spine* test, IVD pressure exhibited an increase from a range of 0.41–0.43 MPa to 0.59–0.66 MPa (Fig. 5a) for the entire range of IVD_1_–IVD_5_. The data closely resembled multiple previous investigations^64–66^ with a maximum discrepancy of 14% as illustrated in the results section. Although such a difference is not significant specifically when considered over the entire range, IVD pressure values fell within normal physiological ranges^64^. Such differences may be directly attributed to the fact that other soft tissues were eliminated from this investigation. Nevertheless, the inclusion of such components, especially the TLF, permitted storing substantial load within these soft tissues. With a smoother transition of loads, suggested by the results of the current test, less pressure is put on the IVDs, and on the spine in total, resulting with more representative intradiscal pressure.

The estimation of IVD pressure from the average normal stress subjected to the IVD surface has been proven to be accurate in the muscle model previously investigated^33^. This is due to the fact that, for thick-walled pressurized structures, the radial stress is equal and opposite to the gauge pressure on the inside surface^69^. However, when dividing the IVD into its nucleus pulposus and annulus fibrosis, the nucleus was modelled as a hydrostatic fluid filled structure. Results for IVD_5_ pressure showed that both procedures resemble each other to a very great extent, with a maximum discrepancy of approximately 4% at 34° flexion (Fig. 5b). Clearly, the second approach provides a more accurate representation of the spinal discs’ biomechanics. However, as in all FE analyses, as long as a model predicts accurate results, approximations to follow the less computationally expensive approach remain applicable.

Overall, results of the intradiscal (IVD) pressure test suggest a validated model of the spinal discs. Combined with the lumbar spine test, both tests suggest a fully validated spine structure, similar to most published spine models which were composed of the vertebral bodies and intervertebral discs. In essence, this lays the foundation to advanced investigations and assessments of low-back pain which has been greatly correlated with IVD pressure^68^.

#### Full spine validation

The final test was done in efforts of concluding on the validity of the full model. However, due to model’s novelties, no previous model that closely resembles the present model was found. As such, the model was first validated against one of the more involved models put forward by Huynh et al.^61^, after which all other soft tissues were included to comment on the full validity of the model.

Initially, applying an increasingly forward flexion on the base model resulted in higher displacements of VBs T_10_–L_5_ (Fig. 6a). It was also noticed that such displacements decrease, until vanishing at the level of the lumbar spine, suggesting the strong support provided by the IVDs. Force–Displacement results were in high agreement with those of Huynh’s up until a force of 350 N. However, Huynh’s investigation showed that vertebral displacements plateau at 350 N, thereafter they start decreasing again, which the present model was not able to predict. This was counterintuitive as, numerically, displacements are believed to ultimately increase with flexion. The results obtained by Huynh’s may be attributed to the adopted coordinate system, from which it seemed that they were measuring displacements in only one direction, and with respect to a fixed coordinate system, rather than updating and measuring the directional displacement. Additionally, their spine model’s excessive movement had exceeded the physiological ranges of static spine flexion, for which they continuously applied flexion until the spine became in a perpendicular position with respect its initial one. Regardless, the base of the present model closely matched their results up to the maximum displacement point with a very small discrepancy of 6% recorded for T_11_ at 300 N force.

With the model accounting for the actual structures of skeletal muscles rather than utilizing vector forces, it was more representative to replicate the flexion movement via muscles contracting. However, reasonable forces should be provided via the muscles which was the reason behind adopting previous muscle data^47^. Such data suggested a maximum flexion position with a total force of 382 N at the T_1_ level, which was slightly higher than the previously used maximum of 350 N, yet was close enough to suggest that muscles are capable of producing accurate spine flexion. Afterwards, all parts were included to investigate the overall effect on vertebral bodies displacements. With the forces presented in Table 3, Force–Displacement results followed the same trend but with a significant drop in vertebral displacements (Fig. 6b). That is, the correlation remained intact but suggested a significant contribution by the other soft tissues. The addition of abdominal pressure from 5 to 36 mmHg played a resistive role, supporting the lumbar spine. Upon investigating such pressure values, they did not seem arbitrary. That is, they compared very well to the IAP values of Mueller’s et al.^70^. Furthermore, as anticipated, the Thoracolumbar Fascia seemed to provide an essential role supporting the spine as well. With the increased amount of flexion, the TLF built an increasing force from 12 to 139 N, resisting the forward flexion motion, and thus, supporting the role of storing sufficient tension to permit the spine to withstand excessive loads. Such findings further support the *intradiscal (IVD) pressure* test where the inclusion of other soft tissues decreases spine flexion, which puts less pressure on the IVDs. Combining all four tests, the model put forth shows accurate and valid results, with the potential of leveraging it to carry out spine-related investigations.

### Sensitivity analysis

Similar to any FE model, verifying the model’s form and results repeatability against input parameters is essential. As such, the guidelines put forth by ASME V&V 40-2018 for numerical models in the biomechanics field were followed to carry out the applicable sensitivity conditions required to conclude on the verification of the model.

Investigating model form was essential to make sure that the modelled parts properly capture the MRI scans upon which the model was based. For this purpose, the best applicable metric seemed to be the volume of each part. Results showed that modelled parts were in excellent agreement with the MRI scans with a maximum difference of 6.17% recorded for IVD_2_ (Fig. 7a). This proves that all parts were accurately graphically modelled with a small margin of error.

Furthermore, investigating the model’s sensitivity against the adopted mesh is crucial as the mesh was one of the model’s novelties. Besides, a common practice in all FE models, by which researchers verify numerical result accuracy, is to run mesh sensitivity analysis. For the purpose of this model, different meshing techniques, both linear and nonlinear, where investigated (Fig. 7b). Results showed a very good agreement, with a maximum discrepancy of 8.7% between the original and the second order tetrahedral mesh. It is worth mentioning that a high reduction in computational time upon adopting the second order mesh for the lumbar model only was observed. Arguably, with an acceptable discrepancy level, leveraging the original mesh brings a great deal of potential, due to eliminating high order of nonlinearities, for the model to be used in medical applications as a quick spine assessment tool or to run implant design optimization, as examples.

Hence, with such acceptable margins of difference, the model can be safely assumed to be robust against critical parameters, leveraging both an accurate valid and repeatable verified representative novel full spine model.

### Limitations

Similar to any in silico model, limitations are inevitable due to the approximation scheme and assumptions made. However, such limitations do not hinder the model’s capabilities as long as the model, with its fixed input parameters, are proven to be valid and accurate towards the content of use that is targeted in subsequent analyses. One of the limitations of the developed model described herein are the material properties and material laws used. Specifically, this investigation lacks a material property sensitivity case-study. Due to the vast amount of parts incorporated, each having a wide range of acceptable material properties, conducting a sensitivity on all possible combinations would be an exhaustive measure. However, since the adopted material properties were previously validated and are adopted from studies against which the current developed model was validated, for which results further proved a valid model, such limitation may not be considered significant.

The application of the model as a spine static stability clinical assessment tool necessitates a balance between accuracy and simulation time cost. Without significant loss in accuracy, besides the novel mesh created for this specific model, the adopted material laws allowed for an extreme drop in simulation time. Although those were mostly linear, given the quasi-static nature of the model, the maximum range of motion simulated still fell within the elastic regime of all components when nonlinearly modelled (hyperelasticity, multiple states interaction, and time effects). Specifically, Fig. 7c shows the maximum strain recorded for the VBs, IVDs, tendons, muscles, and the TLF, which were 0.14, 5.3, 3.5, 9.2, and 2.6%, respectively. Such results were in high agreement with the linear regime of the stress–strain curves of each of those components^25,71–75^; thus, highly supporting the validity of using linear material laws for this range of static motion.

Although the adopted mesh may be considered a smart time saving approach, it can be argued that its implementation reduces the model’s accuracy by a small percentage. However, besides such discrepancy being insignificant, considering all factors, such a meshing technique has a significant application-wise potential. One being the large decrease in computational time, permitting its usage in real-life applications. It presents also a manner in numerical analysis for which redundant nonlinearities may be overcome by such careful meshing.

Lastly, validating the model was a tedious task due to the lack of literature in full-spine modelling. Specifically, the model had to be validated in subsections rather than carrying out a direct validation scheme. Even with such an approach, considerable efforts were carried out to validate the soft tissue section of the model. With literature commonly modelling such effects as force vectors, or even completely eliminating them, multiple studies had to be combined to achieve full spine validation. Even though results showed close resemblance, it may still be argued that this may not be the best approach to achieve validation due to different segregated errors integrated to the final model. Yet, with the obtained results, the authors safely assumed that such anonymous errors were eliminated, concluding with a potentially fully validated model.

### Future work

The capabilities of this model extend beyond numerical modelling and validation. Leveraging such a model may help in various industrial and biomechanical fields from assessing spine injuries, investigating low-back pain, all the way to designing and optimizing medical devices. The authors further admit that this was the first step towards important studies that will be carried out but extend beyond the scope of this paper. One thing to consider first though, is a more comprehensive sensitivity analysis to consider material properties, shell models thicknesses, and further better modelling of other parts if deemed essential.

### Expected contributions

The authors safely assume that this research may contribute in the modelling and biomechanical field. The model introduces the approach of perhaps better modelling biological tissues to fully represent human spine mechanics. The inclusion of the thoracolumbar fascia, abdominal cavity, as well as considering muscle intramuscular pressure in one spine model is, on its own, a novelty. Furthermore, this paper introduces a meshing technique applicable for any complex system that acts as a unitary structure rather than integrating the effects of using complex computations of numerical nodes and elements in contact.

In conclusion, this study developed and validated a novel 3-dimensional volumetric finite element model of the spine including the vertebral bodies, intervertebral discs, major torso muscles, accurate modelling of intra-abdominal pressure, as well as the thoracolumbar fascia. The model was meshed using a new meshing technique that permitted the elimination of redundant nonlinearities involved with contacts computations and greatly accelerated required calculation time. The model was indirectly validated against multiple previously published models in four different validation tests. All test results showed that the model produced reliable results when input parameters were accurately accounted for. Lastly, the model was proven to be robust in light of model form and mesh sensitivity analyses. This novel model provides an accurate method to simulate spine mechanics with the potential of leveraging it for various medical purposes from assessing injuries to designing or evaluating surgical treatments.

## Data Availability

To the best of our knowledge, all data and material are available and transparent as enclosed in the methods and results sections.

## Code availability

To the best of our knowledge, all codes and modelling procedure were clearly described in the “Methods” section to allow for reproducibility of the model. The authors are further ready to disclose and share the model’s codes.
